# Development of a Novel Measure of Informal Caregiver Burnout

**DOI:** 10.1093/geroni/igaa057.1543

**Published:** 2020-12-16

**Authors:** Nicholas James, Daniel Paulson

**Affiliations:** University of Central Florida, Orlando, Florida, United States

## Abstract

Burnout is a concept which has permutated most settings over recent decades. However, due to its roots in occupational research there exists both theoretical and practical gaps to consider when measuring burnout within non-occupational settings, such as informal caregiving. This study developed and validated a measure of burnout for informal caregivers of individuals with Alzheimer’s disease and dementia, the Informal Caregiver Burnout Inventory (ICBI). Theoretical and methodological implications are discussed. Development included a 10-step method for scale development proposed by Boateng and colleagues (2018). Expert feedback on item appropriateness and clarity was collected from 33 caregivers or related professional experts and used to modify the original item-bank. Following this, a national sample of 255 current caregivers was gathered. This survey included the ICBI, two gold-standard measures of burnout, and measures of depression and caregiver burden. Item reduction analysis was used to remove items with poor item-total and inter-domain correlations. The ICBI shows good item-agreement (Cronbach’s alpha= .88) and principles of Item Response Theory were used to measure item- and scale-wide information captured. Convergent validity was then compared against other measures of burnout using Bland-Altman Plots. Divergent validity was similarly assessed by comparing the ICBI to a depression questionnaire. Finally, the predictive validity of each burnout measure was compared to their association with burden and depression. This study suggests that the ICBI may perform adequately as an index of caregiver burnout, and thus is address a methodological and clinical gap in current efforts to understand the dynamics of caregiving.

